# Systemic Complications Secondary to Chronic Liver Disease

**DOI:** 10.1007/s12098-023-04694-7

**Published:** 2023-07-13

**Authors:** Rory Mannion, Emer Fitzpatrick

**Affiliations:** 1 Department of Gastroenterology and Hepatology, Children’s Health Ireland Crumlin, Dublin, Ireland; 2 School of Medicine, University College Dublin, Dublin, Ireland

**Keywords:** Pediatric liver disease, Complications, Malnutrition, Hepatopulmonary syndrome, Portopulmonary syndrome, Cirrhotic cardiomyopathy, Hepatorenal syndrome, Hepatic encephalopathy

## Abstract

The systemic sequelae of chronic liver disease (CLD) may be due to portal hypertension and shunting, malnutrition, and/or a low grade inflammatory state. This article will focus on the consequences of chronic liver disease affecting extrahepatic organs. Portal hypertension underlies many systemic complications of CLD. Aside from varices and ascites, portal hypertension may cause both hepatopulmonary syndrome and portopulmonary hypertension leading to respiratory compromise. Cardiomyopathy may also occur secondary to end stage liver disease. Hepatorenal syndrome is also well recognised and hepatic encephalopathy is a consequence of the effect of liver dysfunction on the brain. Compromise of the immune system is well described in end-stage liver disease leading to sepsis and its consequences. Bony disease including osteoporosis and hepatic arthropathy may both be seen in children with CLD. CLD may be asymptomatic initially but then complications may present as the disease progresses. Furthermore, systemic effects of end stage liver disease may complicate liver transplant. These complications often present insidiously or at the time of acute decompensation. Thus, it is important that healthcare providers are vigilant when caring for children with CLD. This article outlines the secondary complications of CLD with an overview of the definition and diagnosis, pathophysiology, management and prognosis of each.

## Introduction

**Fig. 1:**
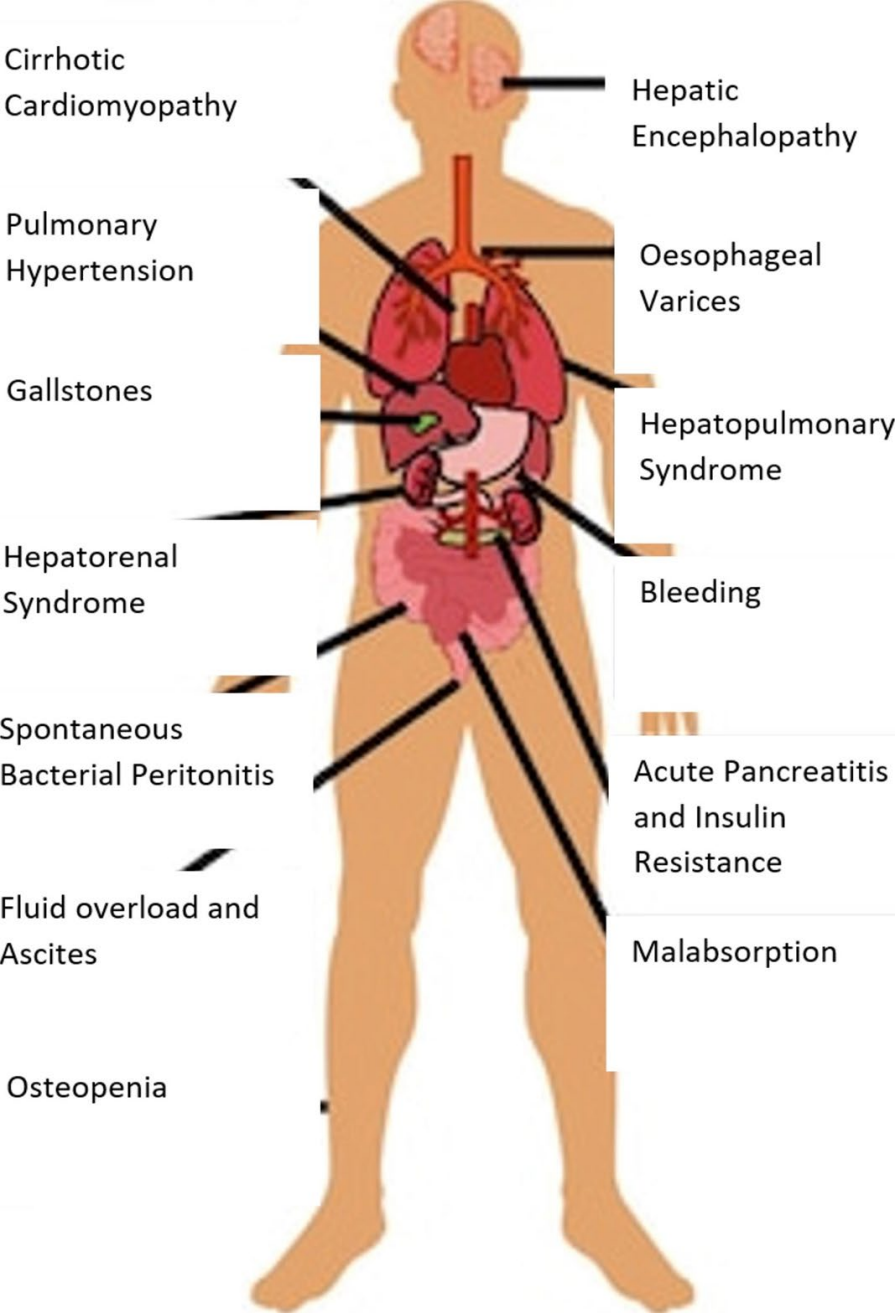
Overview of systemic complications of chronic liver disease

Chronic liver disease (CLD), a consequence of the progressive destruction and regeneration of liver parenchyma leading to fibrosis and cirrhosis, can affect multiple organs. The systemic complications of CLD include nutritional deficiencies, infections, hepatic encephalopathy, hepatopulmonary syndrome, portopulmonary hypertension, hepatic hydrothorax, hepatorenal syndrome, cirrhotic cardiomyopathy, bone and joint disease (Fig. 1).

Portal hypertension is a well-recognised complication, and it underlies several other clinical sequelae. Portal hypertension develops in chronic liver disease in the setting of increased intrahepatic vascular resistance (IHVR). This increase in IHVR is due to structural changes of the architecture the liver in cirrhosis and due to dynamic changes in vascular tone. Increased IHVR also leads to splanchnic vasodilation that further increases portal vein flow and exacerbates portal hypertension [1].

Complications may arise in the context of acute on chronic liver failure, or as an acute decompensation of end stage liver disease [2].

## Malnutrition

### Definition

Malnutrition is a common complication of cholestasis and cirrhosis, leading to an increase in morbidity and mortality [3]. Children with CLD, particularly cholestatic liver disease, often have protein, essential fatty acid, and fat-soluble vitamin deficiencies.

### Pathogenesis

Children with cholestatic liver disease such as biliary atresia have maldigestion and malabsorption of fats. There is often an increase in metabolic demand even in the early stages of disease. With progression of disease, anorexia, poor tolerance of feed, increase in energy expenditure and continuing malabsorption impair growth and development [3].

### Management

The importance of early and regular assessment of nutritional status and requirements of children with chronic liver disease cannot be overstated. Simple measures such as weight and height are mandatory, however in end stage liver disease weight may be unreliable because of ascites and mid-arm circumference is preferred.

Caloric requirements of children with chronic liver disease can be difficult to estimate and are influenced by the underlying disease process and other factors which may be individual to that patient [4].

Medium chain triglyceride (MCT)- enriched formulas are used in cholestatic infants as MCT is directly absorbed into the enterocyte through passive diffusion and unlike long chain triglycerides, do not require bile for absorption. Protein requirements are typically increased due to catabolism and high protein losses.

Adequate intake may not be possible with oral supplements, and enteral nutrition is often indicated, using nasogastric feeds. In some cases, parenteral nutrition is required, particularly in end stage disease. Fat-soluble vitamins need to be supplemented as deficiency is common. Vitamin A deficiency can lead to night blindness. Vitamin E deficiency may present with neurologic symptoms. Vitamin K deficiency impairs coagulation. Vitamin D deficiencies can lead to osteopenia and rickets.

### Outcome

Optimizing the nutritional status of children with CLD by early and frequent assessment of need and by ensuring adequate calorie, protein, fat, and micronutrient provision is crucial in management. Malnutrition in children with CLD negatively impacts survival in addition to development, quality of life, and overall health.

## Infections and Immunity

### Definition

Children with chronic liver disease commonly present with acute decompensation due to infection. Hepatic and non-hepatic infections are more common in children with chronic liver disease with a reported prevalence of 57% in children with decompensated CLD [5].

### Diagnosis

Infections may be bacterial, viral, fungal, or parasitic. The most common infections are spontaneous bacterial peritonitis (SBP), pneumonia, urinary tract infection, and bacteremia. As soon as bacterial sepsis is suspected, broad spectrum intravenous antibiotics should be commenced. Antimicrobial therapies should then be adjusted based on culture results. Enteric gram-negative rods are the most isolated organisms, particularly in SBP. Invasive fungal infections while less common have a high mortality and consequently antifungal prophylaxis is commonly required especially both in the setting of end-stage cholestatic liver disease and in acute liver failure.

### Pathogenesis

There is dysfunction of both the innate and adaptive immune system. This increases the risk of infections and also the likelihood of developing multiorgan failure [6].

### Management

Viral infections are predisposed in liver cirrhosis and can precipitate decompensation. Children should be vaccinated against Hepatitis A and B and receive yearly influenza vaccination. Management of sepsis includes early and judicious use of broad spectrum antibiotics. Paracentesis should be part of the septic work up in a child with ascites and the presence of > 250 white cells/mm^3^ is consistent with a diagnosis of SPB. Broad spectrum antibiotics should be commenced as soon as possible. Cephalosporins are often first line for SBP, however with the rise in multidrug resistant organisms, Pip-tazobactam plus a glycoside may be preferred. Though no guidelines for treatment of SBP in children exist, recent American Association for the Study of Liver Diseases (AASLD) guidelines suggest a duration of 7 d. Secondary prophylaxis is also recommended certain settings in adults using norfloxacin or ciprofloxacin [7].

Treatment of fungal infection needs to be considered particularly in a deteriorating and cholestatic patient as fungal sepsis is associated with increased mortality.

### Outcome

Prognosis is adversely affected, with 25% mortality in one study of children with SPB [8] and with higher in-hospital mortality in those with decompensated CLD with infection vs. those without infection (odds ratio of 3). 

## Neurological

Minimal or covert hepatic encephalopathy is increasingly recognised as a complication of CLD and presents as neuro-cognitive impairment in attention, processing speed, visuospatial perception and integrative functions [9, 10]. A full description of encephalopathy in chronic liver disease will be described elsewhere in this issue.

## Pulmonary

### Hepatopulmonary Syndrome

#### Definition

Hepatopulmonary syndrome (HPS) is a pulmonary vascular complication of liver disease, characterized by the triad of abnormal arterial oxygenation caused by intrapulmonary vascular dilatations (IPVD) in the setting of liver disease, portal hypertension, or congenital portosystemic shunts [11, 12] (Table 1). HPS occurs in approximately 8-16% of children with cirrhosis or severe portal hypertension [13, 14].

**Table 1:** Comparison of the features of Hepatopulmonary syndrome and Portopulmonary hypertension [12]

	Hepatopulmonary Syndrome	Portopulmonary Hypertension
**Definition**	The triad of abnormal arterial oxygenation caused by intrapulmonary vascular dilatations (IPVDs) in the setting of liver disease, portal hypertension, or congenital portosystemic shunts	Portopulmonary hypertension (PPHTN) refers to pulmonary arterial hypertension that is associated with portal hypertension
**Diagnostic criteria**	Criteria 1: Chronic liver disease Criteria 2: A-aDO_2_ ≥15 mmHg or ≥20 mmHg, or ≥ to the age- adjusted value^1^ Criteria 3: Intrapulmonary vascular dilatation at CE-TTE or 99mTc- MAA	Criteria 1: Portal hypertension (15 mmHg, or portocaval gradient >5 mmHg) Criteria 2: mPAP >25 mmHg and mPAOP <15 mmHg mPAP - mPAOP (transpulmonary gradient) >10 mmHg PVR >240 dyn.s.cm^-5^ = 3 UI WOOD
**Severity grading**	Mild: ≥80 PaO_2_, mmHg Moderate: ≥60 and <80 PaO_2_, mmHg Severe: ≥50 and <60 PaO_2_, mmHg Very severe: <50 PaO_2_, mmHg	Mild: 25 mmHg < mPAP <35 mmHg Moderate: 35 mmHg ≤ mPAP <45 mmHg Severe: mPAP ≥45 mmH
**Prevalence**	10%-30% in those with end stage liver disease	6% in those with end stage liver disease
**Pathophysiology**	Both conditions result from a lack of hepatic clearance of vasoactive substances produced in the splanchnic territory.	Both conditions result from a lack of hepatic clearance of vasoactive substances produced in the splanchnic territory.
**Complications**	Intrapulmonary shunts with hypoxemia	Elevated pulmonary pressure and right ventricular dysfunction
**Treatment**	Liver transplant curative in majority in 6–12 mo	Unpredictable outcome

*99mTc- MAA* Technetium macroaggregated albumin lung perfusion scan, *A-aDO*_*2* _Alveolar-arterial oxygen pressure gradient, *CE-TTE* Contrast-enhanced echocardiography, *mPAP* Mean pulmonary artery pressure, *mPAOP* Mean pulmonary artery occlusion pressure, *PVR* Pulmonary vascular resistance

^1^Age-adjusted value: 0.26 (age) - 0.43

#### Diagnosis

Diagnosis is confirmed either using contrast cardiac echography (‘bubble echo’), during which agitated saline microbubbles (>10 microm in diameter) are injected into a peripheral vein. These microbubbles cannot normally pass through the pulmonary capillary bed and therefore cannot circulate to the left heart. Delayed presentation of microbubbles in left heart after 3 or more cycles indicates the presence of IPVDs or a shunt [15]. A false positive may be seen in the case of a septal defect, however in this case microbubbles are seen in the left heart within the first 3 cycles. Quantitative measurement is undertaken using an macro-aggregated albumin (Tc-MAA) lung perfusion scan. Normally these injected particles become trapped in pulmonary capillaries but in case of the presence of shunts they can pass into brain and kidney and are quantified using nuclear imaging. Rarely in pediatrics, arterial blood gas (ABG) analysis P(A-a)O_2_ alveolar – arterial oxygenation gradient as a measure of VQ mismatch is used.

#### Pathogenesis

Pathophysiology of HPS is not yet fully understood. There is a complex interaction between the liver, lungs and gut involving, bacterial translocation, immune cell infiltration of the lungs, nitric oxide and other vasodilators and angiogenesis [15]. HPS results when alterations in the pulmonary microvasculature impair gas exchange. These IPVDs lead to ventilation perfusion mismatch, diffusion restriction and arteriovenous shunts causing impaired gas exchange. Patients may be completely asymptomatic or present with dyspnea and cyanosis. Clubbing, cyanosis and telangiectasia may be the initial signs. Platypnea (dyspnoea when sitting or standing up from lying) and orthodeoxia (>5% or >4 mmHg decrease in partial pressure of arterial oxygen moving from supine to upright) may also be clues to the presence of HPS [16].

#### Management

There is no effective established medical therapy. HPS is a progressive disorder that requires liver transplantation for resolution, though in selected cases control of portal hypertension with other methods has been reported to control the symptoms [17]. Prior to transplantation, treatment is supportive. Patients require long-term oxygen supplementation. Coil embolization may be indicated in palliation. Five year survival post liver transplantation is reported as 76-87% [18, 19], with resolution of HPS in the majority but in some with a prolonged oxygen dependency post-transplant [19].

#### Outcome

The presence of HPS significantly increases mortality and morbidity. In adult patients with HPS, the average survival was 4.8 mo after diagnosis [20].

### Portopulmonary Hypertension

#### Definition

Portal hypertension-associated pulmonary hypertension (portopulmonary hypertension) refers to the presence of pulmonary hypertension in patients with portal hypertension (Table 1). This can be defined as a mean pulmonary arterial pressure >25 mmHg and pulmonary capillary wedge pressure <15 mmHg. The prevalence in patients with cirrhosis is approximately 2% and female patients and those with autoimmune disease are more likely to present with PPH [21].

#### Diagnosis

Portopulmonary hypertension may present as dyspnea on exertion, orthopnea, fatigue, chest pain, peripheral edema or dyspnea at rest. On imaging, a prominent pulmonary artery or cardiomegaly may be appreciated. The pathophysiology is thought to be secondary to increased pulmonary blood flow in the context of portal hypertension which triggers vascular injury and consequent pulmonary vascular remodelling.

#### Management

In mild to moderate cases, liver transplantation is indicated following careful multidisciplinary evaluation of suitability for transplant. Potential medical treatments include prostacyclin analogues (epoprostenol infusion), endothelial receptor antagonists (bosentan) and phosphodiesterase 5 inhibitors (sildenafil).

#### Outcome

The presence of severe portopulmonary hypertension is a relative contraindication to liver transplant and survival is poor at only 14% at 5 y.

### Hepatic Hydrothorax

#### Definition

Hepatic hydrothorax (HH) refers to the presence of a transudative pleural effusion in a patient with portal hypertension who does not have other reasons to have a pleural effusion (e.g., cardiac, pulmonary, or pleural disease) [22].

#### Pathogenesis

Ascites which builds up in the peritoneal cavity moves into the pleural space through small diaphragmatic defects. The majority of hydrothoraces occur in the right pleural space as the right hemidiaphragm is thinner and less muscular. This often occurs in association with SBP and patients with hepatic hydrothorax may develop spontaneous bacterial empyema.

#### Diagnosis

Patients often present with dyspnea, cough and pleuritic chest pain. A thoracocentesis as well as additional imaging (e.g., a chest computed tomographic scan and echocardiogram) should be performed to confirm the diagnosis and to exclude infection or an alternate cause of a pleural effusion.

#### Management

Treatment of hepatic hydrothorax is similar to treatment of ascites. Patients need to restrict sodium and require diuretics. In severe cases patients may require therapeutic thoracocentesis. Ideally leaving in a chest drain should be avoided due to ongoing protein loss and infection. As HH is directly associated with portal hypertension and ascites, transjugular intrahepatic porto-systemic shunt (TIPS) creation to decrease portal pressure may be considered in refractory cases [16].

## Hepatorenal Syndrome

### Definition

Hepatorenal syndrome (HRS) occurs in approximately 5% of children with end stage liver disease [23]. There is no consensus definition in children, so data have been extracted from the adult experience. The International Club of Ascites (ICA) defines HRS using various criteria as the deterioration of kidney function in the presence of advanced liver disease, excluding other causes of acute kidney injury (AKI) including prerenal (hypoperfusion), intrinsic (acute tubular necrosis) and post renal causes [23].

### Diagnosis

Previously, HRS was expressed as type 1 and type 2; type 1 was characterised by a more rapid deterioration in kidney function whereas type 2 was a slower progressive decline of kidney function in the context of advanced liver disease. More recently, the ICA classification of HRS differentiated HRS-AKI and HRS non-AKI (which includes HRS – chronic kidney disease) [23]. The use of absolute values of creatinine to support the diagnosis is less useful in children and in those with poor muscle mass who have a lower total creatinine at baseline.

HRS-AKI may often develop in the setting of ascites or a variceal bleed [23]. The criteria are similar to type 1 HRS: (1) Increase in serum creatinine (sCr) >/= 0.3 mg/dl (>/+ 26.5 umol / L) within 48 h or (2) Increase in sCr >/=50% from baseline occurring within previous 7 d.

HRS non-AKI occurs in the setting of refractory ascites and has a slower progression of dysfunction.

Urinary biomarkers of tubular damage include neutrophil gelatinase associated lipocalin (NGAL) which rises in tubular injury even prior to creatinine rise, kidney injury molecule 1 (KIM-1) interleukin 18 (IL-18) and liver fatty acid binding protein (L-FABP). Blood levels of cystatin C are an early marker of glomerular dysfunction.

### Pathogenesis

HRS is a multifactorial occurrence [24]. Splanchnic arterial vasodilation triggered by portal hypertension together with a hyperdynamic circulation with cardiac dysfunction are important contributory factors. Bacterial translocation and endotoxin production contribute to splanchnic vasodilatation *via* proinflammatory cytokine production. The reduction in systemic vascular resistance activates the renin angiotensin aldosterone system. Vasopressin is released contributing to intense renal vasoconstriction and reduced glomerular filtration rate (GFR). This is a diagnosis of exclusion when other causes of renal failure have excluded.

### Management

Treatment includes fluid expansion and stopping nephrotoxic drugs and excess diuretics when appropriate. Children may have concurrent ascites so fluid management often becomes difficult. There is evidence for the use of 20% human albumin infusions together with frusemide and also for vasoconstrictors including terlipressin [25]. Terlipressin causes vasoconstriction in the arterioles of the splanchnic circulation decreasing portal flow and allowing perfusion of the kidneys. This may be given by bolus injection 6 hourly or by infusion.

In adults, the combination of either terlipressin or epinephrine plus albumin for HRS is recommended. Though there are sparse data supporting this in children, though small pediatric case series suggest that this approach is safe and effective [26].

Renal replacement therapy (RRT) with dialysis may be needed prior to liver transplantation. Continuous RRT removes toxins, helps fluid and electrolyte balance and may serve as a bridge to liver transplant [27]. Simultaneous use of plasma exchange / plasmapheresis and hemodialysis may be required in the setting of encephalopathy and renal dysfunction.

### Outcome

Liver transplant will often reverse much of the kidney injury. It is acknowledged however that overall, approximately 20–40% of children post-transplant will have evidence of kidney dysfunction in any case [28]. In the setting of encroaching kidney failure and the need for a combined or sequential liver kidney transplant may become apparent.

## Cirrhotic Cardiomyopathy

### Definition

Cirrhotic cardiomyopathy is a hemodynamic consequence of portal hypertension. This is characterised by electrophysiological disturbance and systolic and / or diastolic dysfunction, left ventricular hypertension and or QTc interval prolongation [29]. It is typically asymptomatic and latent unless additional stress factors are introduced, such as infection. This can lead to arrhythmias or heart failure.

### Diagnosis

The presence of systolic and diastolic dysfunction on echocardiography with or without electrophysiological abnormalities or elevated biomarkers in cirrhotic patients.

### Pathogenesis

Underlying the complication is myocardial wall stiffness. Hypertrophy occurs due to fibrosis and subendothelial edema.

### Management

In the setting of heart failure due to cirrhotic cardiomyopathy, salt and fluid restriction, diuretics and afterload reduction may be indicated. Afterload reduction in the setting of cirrhosis is challenging due to cirrhotic arterial hypotension.

Cardioprotective drugs have a role in the management of cirrhotic cardiomyopathy though resolution often requires liver transplantation. Cirrhotic cardiomyopathy is not in itself a contraindication for liver transplant, however at time of listing, a multidisciplinary team including cardiologist should discuss perioperative management.

## Bones

### Definition

The prevalence of bony disease, most commonly osteoporosis, in chronic liver disease, ranges from 20-100% depending on patient selection and definition [30].

### Diagnosis

The International Densitometry Society recommendations are based on dual energy X-ray (DEXA) measurements of the lumbar spine and total body less head according to pediatric standards. Z score rather than T score should be reported. DEXA measurements are affected by weight and height as well as age, sex and ethnicity, so an adjusted Z score is recommended [31]. A bone mineral density Z score <2.0 SD below mean is defined as osteoporosis. This refers to a decreased mineral content in the bony matrix. The fracture risk is significant in its presence.

### Pathogenesis

The pathogenesis is multifactorial [32]. Similar to aging related osteoporosis, trabecular bone is lost more rapidly than the cortical bone. Normally, remodelling osteoclasts resorb bone and osteoblasts form new bone, microdefects are repaired during this turnover and stable circulating calcium is tightly controlled by regularising hormones and cytokines. The formation of bone is adversely affected by cholestasis, IGF1 deficiency (normally released by liver in response to growth hormone), hypogonadism, excess mineral deposition in bone, low vitamin D levels, an inflammatory state and sarcopenia. Osteoclast resorption is affected by the inflammatory state and immunosuppressive medications. Osteoporosis is most commonly seen in children with cholestatic disease where fracture risk is prevalent [31].

### Management

As vitamin D may be poorly absorbed in the presence of cholestasis, supplemental vitamin D is almost universally required in chronic liver disease. In children with significant cholestasis, intramuscular injection (sometimes on a monthly basis) is required. Calcium supplements are also sometimes required. In children with autoimmune liver disease or in those post-transplant, the toxicity of long-term steroid use needs to be considered. Exercise is important in maintaining bone health and consideration should be given to this aspect of management in children with CLD [33].

Off label use of bisphosphonates may be indicated in certain scenarios *for example* those with recurrent long bone fractures or vertebral fractures. Various preparations have been used in children including:

Alendronate PO: <30 kg: 5 mg OD (>2 y), 30–40 kg: 5–10 mg OD, >40 kg: 10 mg OD for 24 mo.

Pamidronate IV: 2 mg/kg every 4 mo.

Zoledronate IV: 1–3 y: 0.025 mg/kg 3 monthly, 3–17 y: 0.05 mg/kg (max 4 mg/dose) 3–6 monthly.

### Outcomes

Fractures are common in children with chronic liver disease both pre (10-13%) and post-transplant (12-28%). Vertebral fractures may not be appreciated straight away but can have long term consequences [34]. Liver transplant achieves improvement in bone mineral density 1 y post-transplant, though in the early post-transplant period there may be an increased risk of fracture.

## Conclusions

While CLD is usually asymptomatic initially, as the disease progresses there are extensive extrahepatic complications as outlined. The authors present an overview of the secondary complications of CLD, many of which develop secondary to portal hypertension. Specific therapies may halt the progression and treat the various problems but ultimately patients often require liver transplantation to tackle these complications.
